# Multiple biological characteristics and functions of intestinal biofilm extracellular polymers: friend or foe?

**DOI:** 10.3389/fmicb.2024.1445630

**Published:** 2024-08-19

**Authors:** Fengrong Gong, Shuzi Xin, Xiaohui Liu, Chengwei He, Xinyi Yu, Luming Pan, Sitian Zhang, Han Gao, Jingdong Xu

**Affiliations:** ^1^Department of Clinical Medicine, School of Basic Medical Sciences, Capital Medical University, Beijing, China; ^2^Department of Physiology and Pathophysiology, School of Basic Medical Sciences, Capital Medical University, Beijing, China; ^3^Department of Clinical Laboratory, Aerospace Center Hospital, Beijing, China

**Keywords:** intestinal pathogens, biofilms, exopolymers, biological characteristics, prevention

## Abstract

The gut microbiota is vital to human health, and their biofilms significantly impact intestinal immunity and the maintenance of microbial balance. Certain pathogens, however, can employ biofilms to elude identification by the immune system and medical therapy, resulting in intestinal diseases. The biofilm is formed by extracellular polymorphic substances (EPS), which shield microbial pathogens from the host immune system and enhance its antimicrobial resistance. Therefore, investigating the impact of extracellular polysaccharides released by pathogens that form biofilms on virulence and defence mechanisms is crucial. In this review, we provide a comprehensive overview of current pathogenic biofilm research, deal with the role of extracellular polymers in the formation and maintenance of pathogenic biofilm, and elaborate different prevention and treatment strategies to provide an innovative approach to the treatment of intestinal pathogen-based diseases.

## 1 Introduction

Extracellular polymeric substances (EPS) are a complex structure of biofilms composed of microbial cells, either monospecies or multispecies, exopolysaccharides, proteins, lipids, nucleic acids, eDNA, etc., that adhere to a surface (Toyofuku et al., 2016), which contribute to the unique features of the biofilm lifestyle while also bolstering surface attachment and microbial clustering (Donlan and Costerton, 2002). Currently, it is believed that 99% of bacteria reside as biofilms, with barely 1% existing as planktonic bacteria. In addition, biofilms have been accountable for at least 65% of human bacterial infections (Potera, 1999). Due to the restrictions on mass transfer within the biofilm matrix and the fact that cells within it do not participate in metabolism, biofilm bacteria are particularly resistant to unfavorable conditions including antibacterial agents (Limoli et al., 2015).

The majority of pathogens responsible for gastrointestinal disorders are natural microbes in the gut that create biofilms to adapt to the intestinal environment and cause chronic infection in the host’s gastrointestinal tract. *Salmonella* can form biofilms on the surface of the gut. The surface of the intestine can develop biofilms containing *Salmonella*. These biofilms have the potential to raise antibiotic resistance, which can result in recurrent infections and a higher risk of inflammatory bowel disease, colon cancer, chronic Salmonella disease, and other conditions. Certain pathogenic *E. coli* strains, especially *enterohemorrhagic E. coli* (EHEC) and *enteroaggregative E. coli* (EAEC), can form robust biofilms. These can lead to intestinal diseases such as persistent diarrhea and chronic inflammation. *C. jejuni* biofilms are associated with an increased risk of chronic Campylobacteriosis, post-infectious irritable bowel syndrome (IBS), and inflammatory bowel disease. The biofilm formation of *C. difficile* in the gut can lead to recurrent *C. difficile* infection, antibiotic-associated diarrhea, and pseudomemmembranous colitis. The biofilm formed by *V. cholerae* can contribute to the establishment of persistent cholera infection, enhance environmental survival and transmission, as well as facilitate the production of virulence factors. The probabilities of developing peptic ulcers, gastric cancer, and chronic gastritis is increased once *H. pylori* biofilms are detected (Jandl et al., 2024). Pathogenic bacteria biofilm aid harmful bacteria in evading immune system assaults and increasing antibiotic resistance, hence strengthening their pathogenicity (Grande et al., 2020).

The impacts of extracellular polymeric compounds in enteric pathogen biofilms on virulence and control techniques is a notable field of inquiry within the study of gut microbial ecology. The thick structure of EPS forms a physiological barrier against the entry of antimicrobial drugs, altering pathogen structure, metabolism, and toxicity (Dragoš and Kovács, 2017; Bowen et al., 2018).

Understanding the development of biofilms is crucial for developing innovative techniques to reduce infectious diseases. Starting with the EPS produced during the formation of pathogenic bacterial biofilms, we emphasize the latest breakthroughs in understanding the roles of EPS components like exopolysaccharides, extracellular proteins, and eDNA in the evolution of bacterial biofilms. Key extracellular polysaccharides include Pel, Psl, alginate, PIA, PNAG, and glucans.

## 2 Formation process of intestinal biofilms

**Figure 1:**
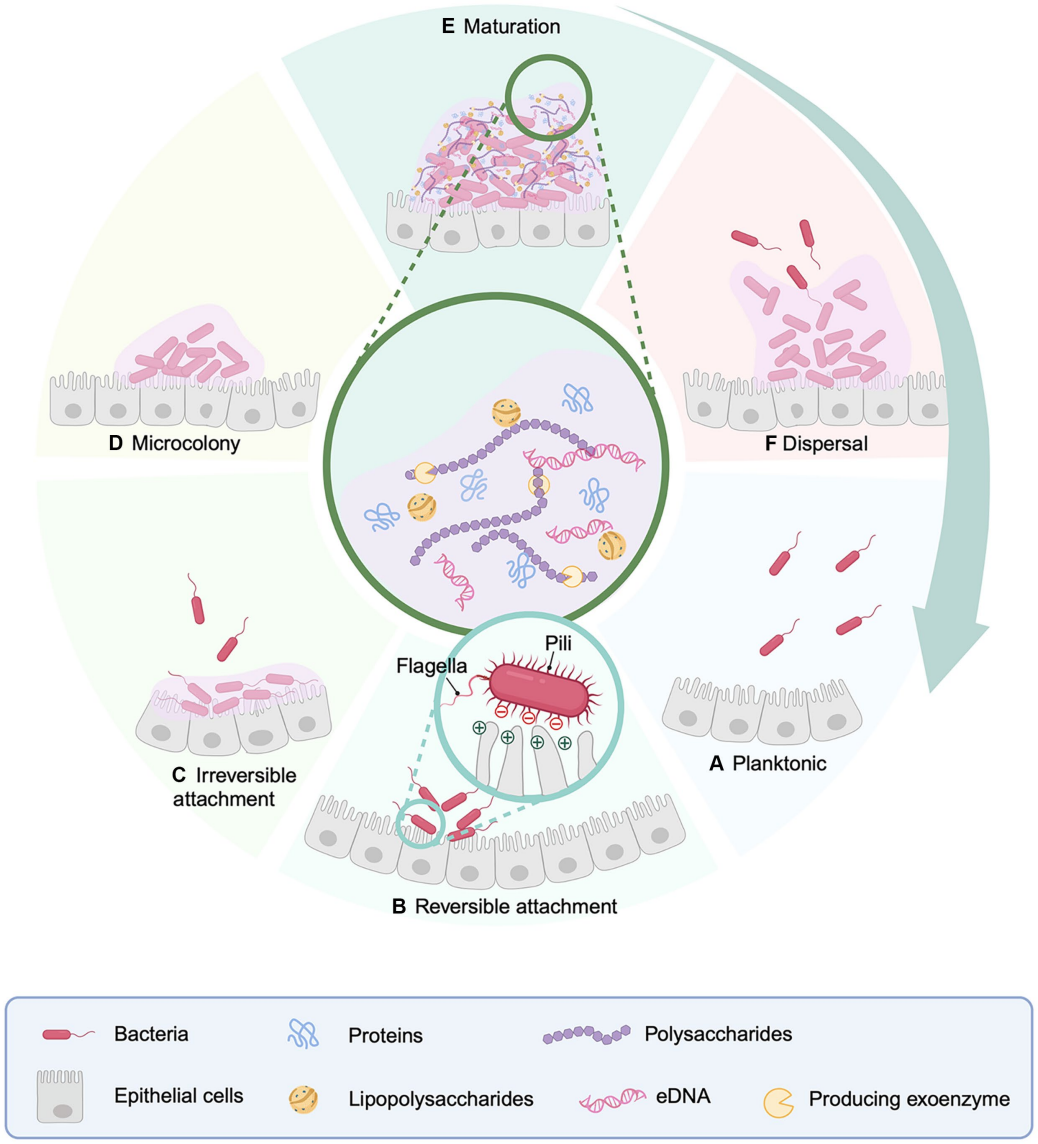
Model diagrams of processes of biofilm formation, development and mechanism. In the intestine, bacterial biofilms build across the intestinal surface on a regular basis. **(A)** Free-floating bacteria on the intestinal surface. **(B)** Reversible bacterial adhesion to the intestinal surface. **(C)** Bacterial irreversible adhesion to the intestinal surface. **(D)** Bacteria begin to multiply, and a tiny quantity of biofilm matrix is produced, resulting in the formation of microcolonies. **(E)** Bacterial proliferation and biofilm maturation. **(F)** Planktonic bacteria are released from the biofilm, seeking new colonization sites on the surface of the epithelial cells. During reversible attachment, the bacterial surface is outfitted with multiple structures like pili (often referred to as fimbriae), and flagella, all of which play a role in mediating interactions with substrates. Additionally, surface characteristics such as negative charge have an impact on the attachment process. Created with BioRender.com.

Bacterial biofilm formation is a multifaceted and dynamic process that primarily encompassing five stages: (i) bacterial reversible adhesion and colonisation, (ii) bacteria irreversible adhesion and aggregation, (iii) generation of microcolonies, (iv) the development and maturation stage of the biofilm, and (v) the detachment and re-colonization stage of bacteria (Figure 1) (Wu Tao et al., 2018). The EPS matrix performs various functions during the stages of biofilm initiation, development, and maturation, closely intertwined with bacterial adhesion, scaffolding, mechanical stability, and protective roles. It immobilizes microbial communities inside biofilm, maintains a series of highly intricate dynamic changes, provides structural mechanical stability, and intricate chemical microenvironments (Karygianni et al., 2020), augments the resistance of biofilm bacteria against antimicrobial agents (Dragoš and Kovács, 2017).

**BOX 1 General characteristics of c-di-GMP.**

Cyclic di-GMP (c-di-GMP) is a critical regulator that governs the growth and dissemination of bacterial biofilms. Diguanylate cyclase (DGC) and phosphodiesterase (PDE) enzymes, that catalyse the production and breakdown of the second messenger, respectively, command cytoplasmic quantities of c-di-GMP, which supervises gene expression, metabolic enzyme activity and biofilm formation via binding to its receptors and allosterically changing the structure and activating the function of its effector. High amounts of c-di-GMP hinder bacterial mobility with increasing EPS matrix production, resulting in biofilm formation.

### 2.1 Reversible adhesion

Surface attachment of bacteria represents the initial and pivotal step in the creation of biofilms, signaling the shift from planktonic to biofilm mode (Karygianni et al., 2020). Planktonic bacteria adhere to the gut surface and subsequently produce EPS, resulting in the formation of a biofilm. When bacteria touch a surface, their interaction is determined by the balance of the forces that either attract or repel them. If the attractive forces outnumber the repulsive ones, the bacteria will stay attached to the surface, otherwise, they will not. Nonspecific physical interactions, including electrostatic forces, hydrophobic interactions, and Lifshitz–van der Waals forces, are mostly responsible for this initial attachment (Dunne, 2002). Gram-negative bacteria can also attach to surfaces via flagella or pili (Laverty et al., 2014). This process is influenced by a number of factors, including ambient conditions, bacterial species, and surface composition (Dunne, 2002). Bacteria utilise flagella to move and overcome the electrostatic repulsion between cell surfaces and host mucosa (O'Toole and Kolter, 1998). The *flgK* gene encoded flagellum-associated hook protein 1 in both *P. aeruginosa* and *E. coli*, with a 40% nucleotide sequence similarity between the two bacteria (Dunne, 2002). Simultaneously, quorum-sensing (QS) causes an elevation in the negative charge on the cell surface, which facilitates bacterial adherence to the surface during the initial phase of biofilm development (Tuson and Weibel, 2013). Individual adhering bacteria are wrapped with a little quantity of secreted EPM at this stage, and the process of biofilm development has not yet commenced. At this point, many bacterial cells may now revert to a planktonic condition, making their attachment reversible (O'Toole and Kolter, 1998).

### 2.2 Irreversible adhesion

Bacteria subsequently establish irreversible adhesion (O'Toole and Kolter, 1998), which is achieved by short-range interactions such as dipole–dipole interactions, hydrogen bonds, ionic bonds, and covalent bonds (Patwardhan et al., 2023). At this stage, surface proteins such as SadB or LapA facilitate in cell-surface attachment (Toyofuku et al., 2016). Despite SadB and LapA are crucial proteins involved in bacterial biofilm formation, not all bacterial species share these proteins. SadB is generally used as an attachment site for the study of *P. aeruginosa* and is conserved in the monomonas. The *Lap* genes are conserved among environmental *pseudomonads* such as *P. putida* KT2440*, P. fluorescens* PfO1 and *P. fluorescens* WCS365, but are absent from pathogenic pseudomonads such as *P. aeruginosa* and *P. syringae* (Hinsa et al., 2003).

*SadB* mutants have been demonstrated the abnormalities during the transition from reversible to irreversible attachment (Caiazza and O'Toole, 2004). According to static biofilm analysis, the biofilm-blocking mechanism of the SadB199 mutant could impede the transition from reversible to irreversible adherence. The SadB199 mutant had few surface-attached cells, except an odd single-cell attachment that did not line with the substrate in the same focal plane as the wild-type strain.

**Figure 2:**
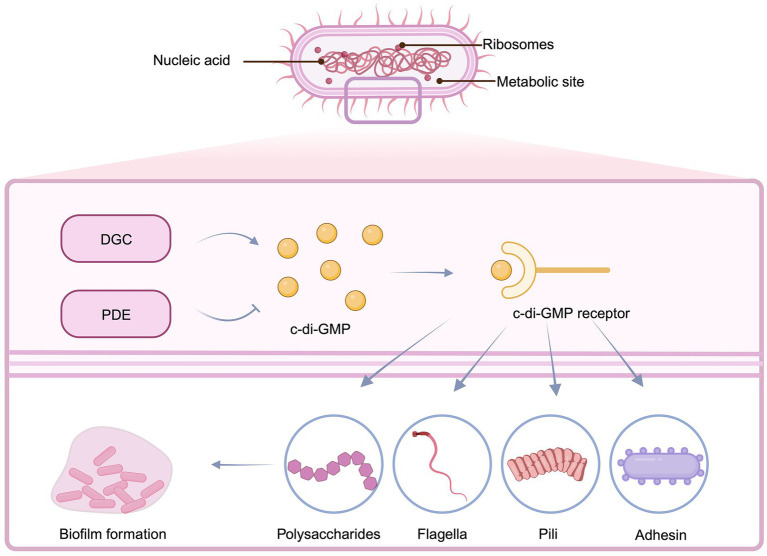
Regulation of the production of EPS components by the synthesis of c-di-GMP. During irreversible attachment, the increase of DGC and the decrease of PDE cause a high concentration of c-di-GMP, which in turn promotes the generation of flagella, pili, exopolysaccharides and adhesin, and enhances the attachment ability. Created with BioRender.com.

Bacterial irreversible attachment is a non-specific approach that relies mostly on interactions between bacterial surface appendages and adhesion factors. Flagella and pili are appendages that have strong affinities with other appendages (Jacobsen et al., 2020). Type IV pili contain sticky ends, which aid in bacterial adherence. Flagella, on the other hand, facilitates bacterial-surface contact by overcoming repulsive forces (Zheng et al., 2021). Fimbriae are produced as a result of c-di-GMP regulation? (See Box 1 for full details), with elevated levels of c-di-GMP fostering fimbriae generation (Figure 2). The GGDEF domain of DGC and the EAL or HD-GYP domain of PDE play important roles in the regulation of c-di-GMP levels in bacteria (Kazmierczak, 2017). Meanwhile, c-di-GMP also modulates flagella, extracellular polysaccharides, and adhesin by activating its receptor. Given irreversible adhesion keeps bacteria from being transported to unfavorable growth settings, making biofilm removal difficult once it has been established. Bacteria commence reproduction creating a tiny quantity of biofilm matrix following irreversible attachment, culminating in microcolonies (Monds and O'Toole, 2009).

Bacteria adhere to the surface by an irreversible change of adhesion mediated by EPS (Rodney M. Donlan, 2001). *P. aeruginosa*, *Bacillus subtilis*, and *Vibrio cholerae* rely on EPS-mediated adhesion to transition from early cell aggregates to microcolonies (Hobley et al., 2015; Hotterbeekx et al., 2017; Wang et al., 2019).

### 2.3 Formation and maturation of biofilms

Following the irreversible adhesion phase between bacteria and surface substrates, the establishment of a biofilm progresses toward maturity. Mature biofilms have highly organized structures that include mushroom-like or mound-shaped microcolonies (Limoli et al., 2015), which are surrounded by a network of numerous channels that allow nutrients, enzymes, metabolic products, and waste materials to be transported (Davies, 2003; Jamal et al., 2018). Bacterial extracellular matrix components have an impact on biofilm maturation (Bucior et al., 2012). EPS is in charge of securing surface adherence, linking cells to form a framework, and sustaining the three-dimensional structure of the biofilm. According to research, EPS components such as eDNA and extracellular polysaccharides promote bacterial aggregation (Das et al., 2011; Oerlemans et al., 2021). Furthermore, the EPS enclosing the biofilm shields bacteria against antimicrobials, host immune systems, oxidation, and metal cations. Under aerobic circumstances, *P. aeruginosa*, primarily guided by its EPS, assembles mushroom-like structures and creates intricate channels within large colonies of rod-shaped cells. Conversely, EPS orchestrates the formation of three-dimensional structures, characterized by interconnected channels and gaps between elongated filamentous cells when exposed to anaerobic conditions. These morphological modifications are crucial in modifying the diffusion characteristics of substances, allowing for more efficient nutrition uptake and waste exchange. Consequently, the structural arrangement of the biofilm dynamically evolves in response to changing environmental conditions, all coordinated by the intricate influence of EPS. The composition and functions of EPS in the major bacterial biofilms of several pathogenic species, such as *P. aeruginosa, E. coli,* and *S. aureus*, are crucial (Table 1). In conclusion, the harmonic interaction of diverse EPS parts contributes to the structural integrity of the biofilm and protects it from environmental stresses (Flemming et al., 2016a,b).

**Table 1:** Main components and roles of EPS in bacterial biofilms.

Bacteria	EPS		Functions	Refs
*P. aeruginosa*	Polysaccharides	Pel	Maintain biofilm establishment and stability; Strengthen intercellular binding; Enhance specific resistance to aminoglycoside antibiotics	Colvin et al. (2011), Friedman and Kolter (2004), Yang et al. (2011)
		Psl	Maintain biofilm establishment and stability; Enhance cell surface and intercellular adhesion; Promote biofilm resistance to antibiotics; Provide protection against bacterial immune response	Ma et al. (2009), Ma et al. (2006), Mishra et al. (2012), Yang et al. (2011)
		alginate	Provide nutrition; Resist harsh environments and protect pathogens from antibiotics and the host’s immune response; Scavenge oxygen free radicals released by neutrophils; Result in a myxoid appearance; Promote immune evasion; Increase resistance to antibiotics	Simpson et al. (1988, 1989, 1993), Govan and Deretic (1996), Sutherland (2001), Leid et al. (2005)
	Proteins	Type IV pilins (T4P)	Adhere to the intestine; Scaffold to stabilize the biofilm; Help bacteria sense the surface of the gut	Kilmury and Burrows (2016), Craig et al. (2019)
		Lectins (LecA/LecB)	Scaffold to stabilize the biofilm; Promote intercellular binding; Adhere to the intestine	Borlee et al. (2010)
		Structural matrix protein		
	eDNA		Promote biofilm formation; Maintain biofilm establishment and stability; Provide nutrition; Increase resistance to antibiotics	Finkel and Kolter (2001), Mulcahy et al. (2008, 2010), Parks et al. (2009)
*E. coli*	Campylopili		Facilitate cell–cell or cell surface interactions and promote biofilm formation Enhance bacterial proliferation and invasion	Flemming et al. (2007)
	Cilium		Mediate biofilm formation, binding and invasion into host cells	Flemming et al. (2007)
*S. aureus*	Polysaccharides	Polysaccharide-intercellular-adhesin (PIA) or poly-β (1–6)-N-acetylglucosamine (PNAG)	Promote the formation of three-dimensional structure of biofilm; Scaffold to stabilize the biofilm; Increase resistance to antibiotics	Bowen et al. (2018), Flemming et al. (2016a,b), Hobley et al. (2015)
	Proteins	Fibronectin Binding Protein (FnBP)	Constitute the biofilm skeleton; Enhance adhesion; Increase resistance to antibiotics	Peacock et al. (2000), Pietrocola et al. (2019)
		Bap	Enhance adhesion; Promote intercellular binding; Maintain biofilm establishment and stability	Anderson et al. (2003), Conrady et al. (2008), Taglialegna et al. (2016)
	eDNA		Promote biofilm formation; Maintain biofilm establishment and stability; Provide nutrition; Repair DNA damage	Das et al. (2014), Finkel and Kolter (2001), Hou et al. (2018)
*Streptococcus mutans*	P1 (Antigen I/II)		Enhance stability; Enhance adhesion; Promotes intercellular binding	Bikker et al. (2002), Nobbs et al. (2009), Sullan et al. (2015)
	Polysaccharides	Glucans/fructans	Enhance the stability of the biofilm scaffold; Promote intercellular binding; Maintain an acidic microenvironment; Antagonize the protective effect of antimicrobials; Provide nutrition	Tanzer et al. (2006)
*Bifidobacterium*	Polysaccharides	DA-LAIM	Enhance the body’s immune response; Antioxidation; Maintain the balance of intestinal flora; Improve intestinal tolerance	Lin and Chang (2000), Bogdan (2001), Kang et al. (2023)
*Lactic acid bacteria*	Polysaccharides	HoPS/HePS	Enhance the body’s immunity; Promotes intestinal health; Antioxidant; Anti-inflammatory	Sato et al. (2004), Cescutti et al. (2013), Pérez-Ramos et al. (2018)

### 2.4 Bacterial biofilm dissemination

Following biofilm maturation, the next and essential phase in the biofilm lifecycle is dissemination. Bacteria migration between planktonic and biofilm phases, in which they cling to multicellular populations (He et al., 2022). As the biofilm evolves, bacteria separate from tiny microcolonies and travel to other surfaces within the gut. The process described above is beneficial for biofilm and EPS proliferation, as the released planktonic bacteria can multiply and form new biofilms. Dissemination serves as a survival strategy for biofilms that involves the detachment behavior initiated by bacteria inside the biofilm in response to particular physiological or environmental alterations, such as tempera- ture changes, nutrient availability, oxygen level, pH and surface type (Abdallah et al., 2014). Transcriptome analyses of *P. aeruginosa* biofilms grown in drip reactors and *E. coli* colony biofilms revealed that biofilm cells respond to environmental gradients by triggering diverse stress responses. This is evidenced by elevated mRNA levels of genes associated with hypoxia (or prolonged anoxia), indicating oxygen deficiency, upregulated expression of *RpoS*-regulated genes, reflecting general stress and stationary phase conditions, and heightened expression of genes related to nutrient stress and slow growth (Abdallah et al., 2014; Lee and Yoon, 2017; Rumbaugh and Sauer, 2020). Dissemination can occur the whole or only a section of biofilm (Rabin et al., 2015).

Dissemination mechanisms are divided into active and passive categories. Passive dispersion is regulated by environmental factors, whereas active dissemination is reliant on cell motility or EPS degradation (McDougald et al., 2011). Bacteria undergo a series of physiologic alterations during active dissemination, including the production of biofilm-degrading enzymes such as glycosidases, proteases, and deoxyribonucleases. These enzymes break down extracellular polysaccharides, extracellular proteins, and eDNA, contributing to the degradation of the biofilm matrix. Sialic acid production, the partial self-lysis of cells within the biofilm, and increased surface cell division are just a few of the physiological changes that bacteria go through during active dissemination (Kaplan, 2010; Petrova and Sauer, 2016; Guilhen et al., 2017; Verderosa et al., 2019). The expression of genes involved in cell motility, such as flagella synthesis and EPS degradation, often increases during the active dispersion phase, whereas the expression of genes involved in EPS formation, attachment, and fimbriae synthesis typically decreases (Kostakioti et al., 2013). The bacterial second messenger c-di-GMP plays a pivotal role in this genetic control. Elevated intracellular c-di-GMP levels have been demonstrated to activate the genes involved in biofilm matrix production and the switch from planktonic to biofilm growth mode (Jenal et al., 2017). Contrarily, a decrease in c-di-GMP concentration enhances bacterial mobility and favors a shift toward planktonic growth, highlighting the importance of c-di-GMP in the transition between the planktonic and biofilm lifestyles (Römling et al., 2013). The evidence indicates that c-di-GMP levels decrease during biofilm dispersal. Reduced intracellular c-di-GMP content increases expression of virulence factors in dispersed biofilm cells, suggesting dispersal is associated with lower c-di-GMP levels (Lin Chua et al., 2017). *E.coli* induces *yhjH* to express PDE, causing a marked reduction in c-di-GMP levels, leading to cell detachment from the biofilm matrix after induction (Thormann et al., 2006). For *P. putida*, the mechanism of detachment caused by reduced c-di-GMP levels is now understood (Römling et al., 2013). Last but not least, the biofilm may extend into the environment, releasing planktonic microorganisms.

In brief, there are various stages to the intestinal growth of biofilm, including reversible and irreversible adhesion, microcolony formation, maturity, and dispersion. The development and ongoing existence of biofilms both depend critically on the generation of bacterial EPS.

## 3 Impact of pathogenic bacterial EPS on the host

Bacterial EPS can have beneficial as well as detrimental impacts on the gut health of the host. While certain EPS can encourage the development of beneficial bacteria and trigger the immune response, others can contribute to the formation of biofilms, leading to chronic infections. To completely comprehend the effect of bacterial EPS on gut health, more experimental evidence needs to be summarized and highlighted.

EPS produced by lactic acid bacteria (LAB) can have several positive effects: LAB-EPS can adhere to intestinal epithelial cells and help restore the impaired intestinal barrier function. EPS from probiotics such as *L. plantarum* can regulate intestinal immunity through pathways such as STAT3 signaling. Some LAB-EPS have antibacterial properties and can inhibit the growth of pathogenic bacteria in the gut (Angelin and Kavitha, 2020). Although the biofilms formed by *S. flexneri* are difficult to eradicate and increase the strain’s resistance to antibiotics, the exopolysaccharides produced by *L. plantarum* can reduce the polysaccharide production in *S. flexneri*’s extracellular polymeric matrix and inhibit the formation of *S. flexneri* biofilms. LAB-EPS could decrease the minimum biofilm elimination concentration (MBEC) of antibiotics against *S. flexneri* biofilm and inhibit *S. flexneri* adhesion to and invasion into HT-29 cell monolayers (Song et al., 2020). Some LAB-EPS have antioxidant properties that may benefit gut health. It’s crucial to remember that EPS effects might vary depending on the circumstance. Probiotics’ EPS usually improve health, but in some situations, any bacteria—even ones that are typically good—may produce too much or the wrong kind of biofilm, which might be harmful.

Considering specific matrix components have been identified as potential virulence agents, EPS plays a substantial part in disease pathogenesis (Peterson et al., 2015; Flemming et al., 2016a,b). Upon bacterial genus and biofilm structure, deviations in variations in EPS composition can contribute to the development of a wide range of ailments. *H. pylori* induced biofilm extracellular protein cag PAI is thought to be closely related to duodenal ulcer, gastric ulcer and gastric cancer (Hathroubi et al., 2018). Excessive EPS synthesis can promote thicker and more resilient biofilms (Colvin et al., 2011), as well as degrade antibiotic molecules by regulating extracellular enzyme production and limiting their effective concentrations around bacteria (Pérez et al., 2012). Furthermore, the EPS matrix can protect pathogens from host immune responses (Andersen et al., 2021). Biofilm EPS also enables pathogens to adhere firmly to surfaces, making infection more difficult to eradicate (Zhao et al., 2023).

### 3.1 Impact of polysaccharides on gut health

Water-soluble polysaccharides produced by certain bacteria as part of growth metabolism are known as extracellular polysaccharides, which, also referred to as secondary metabolites, are crucial for bacterial growth (Schmid, 2018). One of the processes involved in the creation of bacterial biofilms is the production and activity of polysaccharides. Given the high biological activity of many bacterial polysaccharides, it’s probable that these polysaccharides are bacterial responses to antimicrobial compounds prevalent in the environment.

Polysaccharides can increase bacterial toxicity by promoting bacterial adhesion to host cells, enhancing pathogenic bacteria resistance to host immune responses, and modifying virulence expression. Polysaccharides, particularly poly-N-acetylglucosamine (PNAG), have been found in multidrug-resistant *S. aureus* biofilm matrices. In order to promote bacterial survival and spread, PNAG creates fibrous structures on the surface of bacteria to enhance bacterial survival and dissemination, which increase biofilm aggregation and stability while blocking host immune responses and interfering with inflammation and phagocytosis (Burgui et al., 2018). Mutations in PNAG can diminish neutrophil recruitment within the peritoneal cavity and compromise the body’s bacterial clearance capabilities (Ferreirinha et al., 2016). Psl and Pel within *P. aeruginosa* EPS can enhance biofilm stability and density while lowering antibiotic permeability, reducing the antibiotic efficacy against bacteria (Zegans et al., 2012).

The three primary EPS produced by in *E. coli* are Poly-β-1,6-N-acetyl-d-glucosamine (PGA), cellulose, and capsular polysaccharides antigen (Sharma et al., 2016). By mediating intercellular adhesion and attachment to the gut surface and acting as a stabilizer, PGA encourages the formation of biofilm. Cellulose synthesis is pivotal for mature biofilm formation (Uhlich et al., 2014). Capsular polysaccharides form a protective envelope around bacterial cells, shielding them from specific environmental conditions (Vogeleer et al., 2014).

In addition, a variety of proteins interact with exopolysaccharides to form structural and functional complexes (Ryder et al., 2007), which can also cause bacteria to aggregate and form biofilms to become more stable, which makes it more difficult for bacteria to be removed by the human immune system, leading to many chronic diseases.

### 3.2 Effects of extracellular proteins on the stability of EPS

Bacterial biofilms are formed through a series of intricate stages in which proteins provide support and structural frameworks, upon which aggregate bacterial cells using interactions and mutual attraction, shaping a sturdy biofilm structure (Karygianni et al., 2020). Proteins across the biofilm matrix can facilitate bacterial adhesion to host cell surfaces (Kostakioti et al., 2013), allowing for collective behavior and increasing pathogenicity via intercellular signaling and cooperative activities (Rutherford and Bassler, 2012). Extracellular protein may also penetrate into host epithelial cells, interfere with the cell signal transduction pathways, and minimize the host’s immune response, thereby enabling them to evade the host immune attack (Hatakeyama, 2014).

Surface Protein A (SpA) has been identified in the biofilm matrix of *S. aureus* (Speziale et al., 2014). Protein A-IgG complexes arise as a result of the interaction between Protein A and IgG and precipitate out of the aqueous solution. This interaction occurs via SpA’s strong binding to the Fc region of IgG molecules, which has implications for immune responses and bacterial evasion strategies. For example, SpA can inhibit the collective immune response during *S. aureus* bloodstream infection by cross-linking B cell receptors (IgM)(Shi et al., 2021). SpA can also limit complement activation and promote bacterial evasion by interfering with IgG hexamer formation. Also, protein A-IgG complexes can cause immune complex deposition as well as the activation of inflammatory responses (Palmqvist et al., 2002). This can exacerbate the pathogen infections.

Amyloid-like fibers have been identified as contributing to maintain the biofilms structural integrity by acting as scaffolds for the biofilm matrix and providing mechanical stability. Functional amyloid fibers are a synonym for Fap fibers. The presence of the fap operon, which includes multiple genes such as *fapA*, *fapB*, *fapC*, *fapD*, *fapE*, and *fapF*, has been uncovered to contribute to biofilm production in *P. aeruginosa* and other *Pseudomonas* species. Fap fibers are amyloid-like fibers made primarily of FapC and smaller subunits such as FapB and FapE (Erskine et al., 2018).

The shedding of villous-like epithelial cells is a crucial component of intestinal dynamic equilibrium and is regarded as a natural defense mechanism that shields the intestines from the adhesion and colonization of pathogens. Impeding the detachment of epithelial cells, on the other hand, strengthens the adherence and settlement of pathogens on the gut surface. Extracellular proteins from specific bacteria can minimize epithelium shedding and hence improve biofilm adherence. Notably, *H. pylori* injects CagA protein, a type IV secretion system effector molecule, into epithelial cells, inhibiting cell apoptosis and thereby enhancing its colonization within the stomach (Fischer, 2011). Similarly, *Shigella* employs its type III secretion system effector protein OspE to strengthen the adhesion between epithelial cells and the basement membrane, impeding epithelial cell shedding and augmenting bacterial adhesion and colonization (Hu et al., 2021).

As a whole, the extracellular proteins in EPS play a crucial role in mediating biofilm adherence to surfaces. They promote interactions within the biofilm and the epithelial cells, aid in the formation of the EPS matrix, and improve the biofilm’s adhesion to the substrate. Recognizing the roles played by these proteins can give a deep understanding of biofilm adherence and potentially contribute to the discovery of biofilm management strategies.

### 3.3 eDNA involved in the gut homeostasis

Extracellular DNA (eDNA) is the biofilm matrix component that is essential for bacterial biofilm formation, development, structure regulation, and function. eDNA is abundant in both clinical and environmental settings, providing a large pool of genetic material that can be accessed by naturally competent bacteria (Nolan et al., 2020). So far, the specific mechanism of action of eDNA is not fully understood, but scientists have found that they are widely present in biofilms, it would form molecular complexes and interact with a multiplicity of chemical substances, such as biofilm exopolysaccharides, biofilm proteins, inflammatory cells and membrane vesicles. eDNA would not simply accumulate in the EPS. Instead, it will generate a sort of crosslinked auto-setting gel with heterogeneous features, undergoing progressive modifications, and remodeling during different phases of biofilm maturation (Campoccia et al., 2021). Biofilms provide an ideal environment for natural transformation due to the high concentration of eDNA and the close proximity of cells. For example, *P. aeruginosa* produces a large number of eDNA required for biofilm formation and has been found to promote the natural transformation of genomic and plasmid DNA within biofilms (Nolan et al., 2020). eDNA can facilitate the spread of antibiotic resistance genes among bacteria in biofilms. A study demonstrated that live donor *V. dispar* cells could transfer the conjugative transposon Tn916 to four different Streptococcus species recipients in a multispecies oral biofilm (Hannan et al., 2010). Studies suggest that eDNA is a vital component of *C. albicans* biofilm EPS, which enhances adhesion, strengthens biofilm integrity by interacting with other components, and limits antifungal medication penetration, thereby building treatment resistance (Martins et al., 2010). Furthermore, by joining DNA chains via base pairing or interacting with other chemical components, eDNA may establish nanowire structures, resulting in a stable network structure that connects bacterial cells, interacts with other cells matrix molecules as well as improve biofilm stability (Flemming and Wingender, 2010), allowing pathogenic bacterial to survive and replicate in the host. eDNA can generate resistance to antimicrobials by increasing target mutations in bacteria and reducing medication transport and penetration into the biofilm interior (Saqcena et al., 2013). In addition, certain pathogenic bacteria produce eDNA to suppress host immune responses, allowing them to survive and replicate (Bialas and Permar, 2016). Taken as a whole, eDNA is a critical component of biofilm matrices. It plays a structural role in the intricate architecture of bacterial biofilms. More study is needed to fully understand the relationship between eDNA and EPS in biofilms, as well as the implications for infection and disease.

In summary, depending on the specific EPS and the context of the interaction, probiotics such as *Lactobacillus, L. plantarum, Bifidobacterium* EPS can improve the intestinal barrier function, regulate the intestinal immune response, inhibit the growth of pathogenic bacteria, antioxidant to beneficial intestinal health. Pathogenic bacterial such as *S. aureus* and *P. aeruginosa* EPS can exert detrimental effects on the host by enhancing pathogen adhesion, improving biofilm stability, inhibiting host immune responses, and impeding antibiotic penetration, ultimately leading to chronic infection.

## 4 Strategies to prevent and treat the production and development of pathogenic EPS

It would be more helpful to understand a variety of extracellular polymeric substances (EPS) that are found in the microbial communities of the human gut in order to investigate the mechanisms underlying EPS characteristics and to manage or prevent pathogenic organisms from producing EPS. The variety of EPS generated by various pathogenic bacteria provides considerable hurdles in appropriately treating their harmful effects on the host. The types of EPS produced by pathogenic biofilm-forming pathogens mainly include exopolysaccharides, extracellular protein, eDNA. Exopolysaccharides include *P. aeruginosa*’s polyanionic exopolysaccharide (Alginate), *S. aureus* and other pathogens’ polycationic exopolysaccharide (PNAG), another polycationic exopolysaccharide (Pel), a homopolysaccharide found in some bacterial biofilms (Cellulose), and a polysaccharide produced by *Pseudomonas* species (Psl). Extracellular proteins include fibronectin binding protein (FnBP), Bap, type IV pilins (T4P), lectins (LecA/LecB), structural matrix protein, surface protein. Unlike bacterial cells that are tightly entrenched within biofilm colonies, EPS is visible to the outside world. Furthermore, EPS has a porous structure by nature, its biofilms properties make it a viable target for anti-biofilm therapy. Dispersing biofilm colonies makes bacterial more vulnerability to antimicrobial drugs and host defenses (Izano et al., 2009). A multi-targeted or combination treatment is usually necessary to fight the toxicity of pathogenic bacterial EPS. Targeted treatment can be done by decreasing EPS synthesis, inhibiting adhesion mediated by adhesins, or eliminating existing EPS inside the biofilm (Xavier et al., 2005). A variety of intracellular and extracellular signaling networks, as well as non-signaling mechanisms, govern the synthesis of biofilm EPS (Koo and Yamada, 2016; Dragoš and Kovács, 2017). Controlling of biofilm formation represents a crucial avenue for treating chronic inflammatory and infection-related diseases.

### 4.1 Inhibition of pathogen adhesion in the intestinal tract

EPS as the micro environment of bacteria composition, provides stable basis for the survival of bacteria. It can reduce the influence of external environment on bacteria and is conducive to the growth and reproduction of bacteria. The results brought by EPS are related to the species of bacteria. The beneficial bacteria (such as *Bifidobacterium, Lactobacillus, Bacillus subtilis*) by producing EPS increase biological membrane structural support, protect bacteria, provide nutrition for the bacteria, and a beneficial effect on the human body; EPS from pathogenic bacteria (such as *Vibrio cholerae, Salmonella, S. aureus, P. aeruginosa, S. pneumoniae*) can increase resistance to antibiotics, and transmission of virulence factors leads to persistent infection. Therefore, when preventing pathogenic EPS from attaching to the intestinal surface, the protection of beneficial bacteria should be considered.

Pathogenic bacteria produce distinct signaling molecules during the manufacturing process for controlling EPS synthesis and secretion. Disrupting these signal transduction pathways, such as targeting the synthesis enzymes or receptors of signaling molecules, could prevent the development and adhesion of bacterial biofilms in the intestines (Das et al., 2020), providing another option for innovative therapeutic approaches and the detailed illustration shown in Box 2.

**Figure 3:**
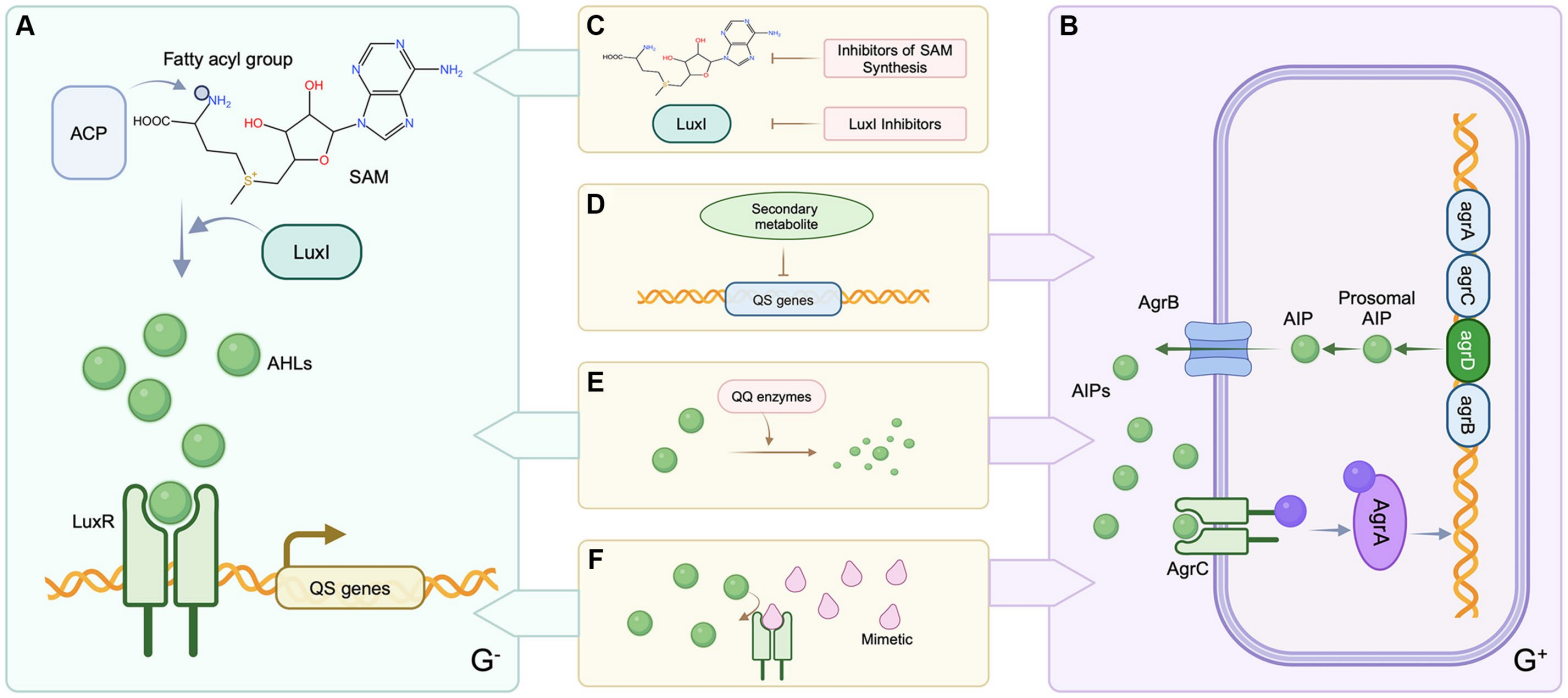
Patterns of QS inhibition mechanisms in Gram-negative and Gram-positive bacteria. **(A)** QS systems in Gram-negative bacteria. (**B)** QS systems in Gram-positive bacteria. **(C)** Inhibition of SAM synthesis or reduction of AHLs synthetase activity. **(D)** Blockade of QS gene expression by microbial secondary metabolites **(E)** Degradation of signaling molecules. **(F)** Signal molecule analogs competitively inhibit the binding of signal molecules to receptor proteins. Created with BioRender.com.

Quorum sensing inhibitors are a type of chemical that disrupts the development of bacterial biofilms, the generation of virulence factors, or the expression of harmful genes by targeting bacterial quorum sensing systems. Unlike conventional antibiotics, these inhibitors specifically decrease the harmful features of bacteria, reducing the formation of drug-resistant mutants while without impacting bacterial growth. Quorum sensing inhibitors employ various mechanisms to prevent the production of bacterial biofilms. To begin, the formation of biofilm is prevented by inhibiting the synthesis of signaling molecules (Figure 3) (Li et al., 2020). S-adenosylmethionine (SAM) and acyl carrier protein are used as substrates in the synthesis of AHL (N-acyl homoserine lactone). Consequently, inhibitors synthesized from SAM can serve as quorum sensing inhibitors for N-acyl homoserine lactone (AHL). AHL production can also be disrupted by decreasing the activity of an enzyme involved in AHL synthesis (Kalia, 2013). Secondly, signaling molecule enzymatic degradation is a critical phase and plays a blocking role in the QS system of both gram-negative and gram-positive bacteria. Once synthesized, signaling molecules can be hydrolyzed by degrading enzymes, and these enzymes prevent signaling molecules from reaching critical threshold concentrations. As the QS system cannot perceive the signaling system, this hindrance the QS system’s regulation of bacterial expression (Brackman and Coenye, 2015). Third, current research predominantly focuses on developing analogs of natural AHL signaling molecules. This is accomplished mostly by altering the acyl side chain or lactone moiety of AHL. These analogs competitively bind to the active site of specific receptor proteins, ultimately impeding microbial QS systems (Brackman et al., 2012). Meta-bromo-thiolactone (mBTL), an analog of AHL, induces conformational changes in the LuxR receptor, weakens its interaction with RNA polymerase, reduces LuxR receptor transcriptional activation potential, and thus inhibits the expression of virulence factor genes in *P. aeruginosa*, preventing biofilm formation (O'Loughlin et al., 2013). For gram positive bacteria, microbial secondary metabolites can inhibit the expression of QS related genes, block the QS system of pathogens, inhibit the expression of a variety of virulence factors, and significantly inhibit the formation of biofilm. L-Phe-L-Pro and L-Tyr-L-Pro, two cyclic dipeptide compounds produced by *Lactobacillus reuteri*, can inhibit the expression of various exotoxins of *S. aureus* and significantly prevent the development of intestinal inflammation (Li et al., 2011). In addition, an important finding in studying AIP signaling molecules has the ability of QSIs AIP analogs to competitively inhibit the QS process. These analogues can adversely affect to combined with the receptor proteins, thus inhibiting QS process (Tal-Gan et al., 2016). Based on the available studies, the value of QSIs is a feasible option for future evolution of innovative anti-enteric pathogens.

**BOX 2 Concept and process of Quorum sensing system.**

Quorum sensing is a process employed by bacteria for communication and coordination of mutual behaviors and activities that regulate many physiological processes. Autoinducers (AIs), which serve as signaling molecules, are synthesized and secreted throughout the bacterial development process. AIs accumulate in the surrounding environment as bacterial density grows. When AIs reach a crucial threshold, they influence gene expression by attaching to receptors and either blocking or activating them, so influencing key biological activities. This is referred to as bacterial quorum sensing. N-acyl homoserine lactones (AHLs) are the most common AIs used by Gram-negative bacteria. AHLs are produced by LuxI family proteins that use S-adenosylmethionine (SAM) and acyl carrier protein (ACP) as substrates. This entails moving fatty acyl derivatives coupled to acyl carrier protein. Transferring fatty acyl derivatives bound to acyl carrier protein to the amino group of SAM for synthesis is involved. When AHL concentration reaches the critical threshold, AHL interacts with LuxR family DNA-binding receptor proteins, forming the AHL-LuxR complex then attaches the gene promoter region on DNA, initiating the activation of downstream genes.

Different from gram-negative bacteria, gram-positive bacteria mainly use autoinducer peptide (AIP) as the signal molecule of QS system, which is transferred by ATP-binding cassette (ABC) system or membrane channel proteins (Kalia, 2013). When the concentration of AIP in the environment reaches a certain threshold, it binds to the two-component signal transduction system autoinducible peptide receptor on the cell membrane, and histidine protein kinase is activated. The histidine residue in the kinase is phosphorylated, and the phosphate group is further transferred to the receptor protein through the aspartic acid residue (Guo and Sun, 2017). The Agr system of *S. aureus* has been widely studied. In SA, *agrD* encodes the autoinducible peptide precursor peptide AgrD, which is processed by AgrB to secrete AIP to the extracellular. *agrC* encodes the histidine protein kinase AgrC, which together with agrA, encoded by AgrA, forms a two-component signal transduction system. AgrA can bind to the promoter P2 and P3 to regulate the transcription of RNAII and RNAIII, respectively. RNAII encodes four open reading frames: *agrB*, *agrD*, *agrC* and *agrA*. *agrD* produces AIP precursors, which are processed and secreted by AgrB to form AIP. AIP binds to the transmembrane receptor AgrC and triggers phosphorylation of the two-component system, causing AgrA phosphorylation. AgrA binds to the promoter sites of RNAII and RNAIII, in which RNAIII induces the expression of virulence genes, and RNAII further promotes the synthesis of AIP, forming a positive feedback loop (Reuter et al., 2016).

### 4.2 Degradation and disruption of EPS

Bacterial resistance mechanisms are commonly associated with the bacterial aggregation producing biofilms, establishing a natural barrier that hampers antibiotic penetration, and the evolution of bacterial resistance, particularly when resistance occurs concurrently at the cellular and population levels. EPS, such as polyβ-1, 6-n-acetylglucosamine (PNAG), nucleic acid, PGA, Pel and Psl exopolysaccharide, generation formation in bacterial biofilms is influenced by a variety of extracellular/intracellular signal networks and non-signaling mechanisms. Disrupting these pathways effectively regulates the production of bacterial biofilms.

#### 4.2.1 Matrix-degrading enzymes degrade EPS

Matrix-degrading enzymes can serve as a valuable tool for investigating both the composition and functionality of the biofilm matrix. These enzymes contain a tendency to depolymerize and break down the components of the EPS matrix, resulting in active biofilm dispersion. One strategy involves includes incorporating specific enzymes to improve the breakdown or disruption of specific components inside EPS. By decreasing or limiting biofilm formation and stability, this approach reduces biofilm synthesis.

**Figure 4:**
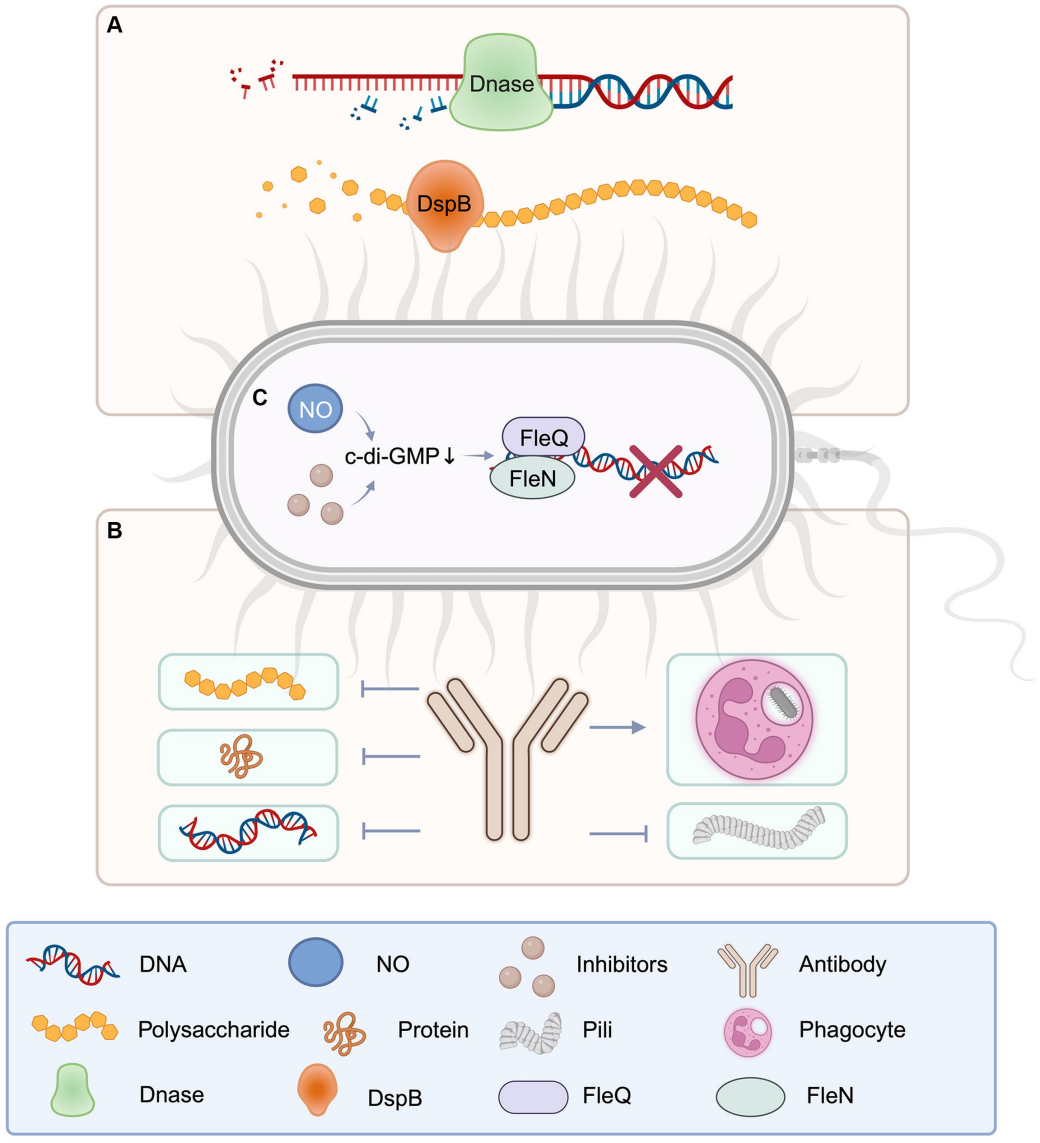
Schematic diagram of catabolic enzyme, antibodies, and c-di-GMP involved in EPS degradation. **(A**) DNase for extracellular DNA and disperse B for extracellular polysaccharide, as important enzymes to degrade EPS components, play an important role in the destruction of pathogenic biofilms. **(B)** Antibodies against EPS components can be targeted to block the production of polysaccharides, proteins, DNA, and fimbriae, and promote phagocytosis of bacteria. **(C)** The reduction of c-di-GMP will affect the gene expression of EPS components, thus hinders the production of EPS. Created with BioRender.com.

Several enzymes and enzyme mixtures have demonstrated anti-biofilm activity both *in vitro and in vivo*, with deoxyribonuclease I (DNaseI) and dispersin B (DspB) being two noteworthy examples (Figure 4) (Kaplan, 2009). DNaseI degrades eDNA. DspB, on the other hand, destroys poly-β-1,6-N-acetylglucosamine (PNAG), which is derived from the bacterial membrane. PNAG is an extracellular polysaccharide that, together with proteins and eDNA, firmly envelops biofilm matrix, providing a substantial structural component essential for adhesion, immunogenicity, cellular protection, and resistance.

In flowing cells, DNaseI inhibits the production of *P. aeruginosa* biofilms (Whitchurch et al., 2002). Biofilm development was prevented when *P. aeruginosa* was seeded in culture medium with DNaseI. According to subsequent study, DNaseI shows anti-biofilm activity against a variety of gram-negative and gram-positive bacteria. The action of DNaseI is believed to restrain the formation of biofilms by breaking down nucleic acids located on the surface that act as adhesion molecules, thereby diminishing the initial attachment of bacterial cells (Qin et al., 2007). DNaseI has a novel way to degrading eDNA, which contributes to intercellular adhesion within biofilm colonies.

Dispersin B (DspB) is a 42 kDa hexosaminidase enzyme associated with facilitating cell detachment and dispersion from biofilm colonies. The breakdown of PNAG polymers inside the biofilm matrix is involved in this process (Kaplan et al., 2003). DspB is commonly used to degrade PNAG on bacterial surfaces to disrupt biofilm structures. For the influence of intracellular PNAG may be relatively small. DspB has been revealed to impede biofilm formation by several gram-negative and gram-positive PNAG-generating bacteria (Itoh et al., 2005), as well as isolate established biofilms (Kaplan et al., 2004). The main difference between targeting extracellular PNAG and targeting intracellular PNAG lies in their different biological functions and locations of action. Targeting extracellular PNAG is of advantage because extracellular PNAG is the main component of biofilm. Targeting PNAG can effectively destroy the biological membrane structure, rendering the bacteria more susceptible to treatment interventions, such as the effect of antibiotics or the removal by the host immune response. In contrast, the challenge of targeting intracellular PNAG is that intracellular PNAG is usually not directly involved in biofilm formation, so targeting intracellular PNAG may have other effects on the bacteria themselves and be extremely weak on the biofilm. Therefore, targeting strategies against extracellular PNAG may be more directly effective as it directly affects biofilm formation and stability, while targeting intracellular PNAG may require more in-depth investigation and careful consideration of its potential effects (Wang et al., 2023). DspB has additionally been demonstrated to enhance the susceptible of biofilm bacteria to antibiotic killing. Treatment of *Pleuropneumoniae* test-tube biofilms with ampicillin in the absence of DspB resulted in a reduction, which was an inversion in the presence of DspB (Izano et al., 2007). DspB sensitizes biofilms not only to chemical agents but also to biological agents such as phages and macrophages. Phages lower the amount of bacterial biofilm cells by roughly two orders of magnitude more efficiently than nonenzymatic phages when employed to treat *E. coli* biofilms. Additionally, matrix-degrading enzymes have been demonstrated to be effective as anti-pathogenic bacterial biofilm formation agents (Lu and Collins, 2007).

In addition to the two matrix-degrading enzymes mentioned above, several other matrix-degrading enzymes have been identified in enteric pathogens and disease-related symbionts, such as proteases, including metalloproteinases (M18 and M12B families), trypsin, papainase and calpain; carbohydrate-active enzymes (CAZymes), including α-mannosidases, β-galactosidase, β-glucosidases, chitinases and N-acetylglucosamine deacetylases; specific EPS-degrading enzymes, including alginate lyase and DNase I. The enzymes produced by various gut microbes, including bacteroides (*B. fragilis, B. ovatus, B. theta, B. vulgatus*), *Ruminococcus gnavus, Prevotella copri* (Porras et al., 2022). Matrix-degrading enzymes promote intestinal disease progression through the direct degradation of EPS components, including collagen, laminin, and fibronectin, as well as the breakdown of glycosaminoglycan and glycoprotein in the intestinal EPS. At the same time, it can destroy the protective biofilm layer, which may increase the susceptibility to pathogens. The efficacy of antibiotics can also be enhanced by increasing penetration into biofilms (Torelli et al., 2017).

A greater amount of research is needed to investigate and optimize the use of matrix-degrading enzymes for biofilm management, considering the biofilm’s unique properties as well as the intended outcome.

#### 4.2.2 Antibodies targeting specific EPS components

There is no uniform method for extracting all EPS components, which have an impact on EPS component detection. Currently, antibodies targeting EPS components are only available for immunization against removed sections. ESKAPEE pathogens are the seven most common opportunistic intestinal pathogens, including *Enterococcus faecium, S. aureus, Klebsiella pneumoniae, Acinetobacter baumannii, P. aeruginosa, Enterobacter SPP* and *E. coli*. They are highly resistant and cause more than 3.5 million deaths worldwide each year. HuTipMab, a human monoclonal antibody against bacterial DNA-binding proteins (DNABII), which are widely present in the biofilms of ESKAPEE pathogens and other pathogens such as *Salmonella typhi*, was designed to destroy bacterial biofilms and reduce their resistance to antibiotics (Devaraj et al., 2021). Monoclonal antibodies against EPS derived from *P. aeruginosa* have been shown to include Psl-binding epitopes (DiGiandomenico et al., 2012), which has been demonstrated to function as a non-serum-dependent antigen, and the presence of Psl-specific antibodies enhances the ability to kill *P. aeruginosa* through phagocytosis, inhibits epithelial cell adherence, and provides prophylactic protection in animal models of *P. aeruginosa* infection (Pier et al., 1994; Pier, 2007; Jones and Wozniak, 2017). Furthermore, antibodies produced by immunization against the *Enterococcus faecalis* pilus tip protein EbpA effectively hinder bacterial adhesion to fibrinogen and the biofilms formation (Flores-Mireles et al., 2014). Immunization and screening elicited antibodies against PGA in *E. coli* biofilms indicating an interaction with PGA and inhibition of biofilm formation (Dörsam et al., 2016). Similarly, research corroborates that the polysaccharide PNAG is a prominent component of *S. aureus* biofilms, with monoclonal antibodies targeted to block PNAG synthesis and *S. aureus* biofilm formation. Meanwhile, anti-DNABII (Dorman, 2009; Devaraj et al., 2018) and anti-type IV pilin antibodies (Novotny et al., 2015) have been revealed to impede EPS components.

The ability of antibodies to penetrate and disperse biofilms is a critical issue. The dense and complex structure of biofilms can hinder the penetration of antibodies, especially in mature biofilms (Jiang et al., 2020). To enhance penetration and dispersion, antibodies are often used in combination with other treatments. For example, combining antibodies with enzymes that degrade EPS components, such as glycoside hydrolases, can ameliorate biofilm destruction and increase antibiotic efficacy (Jiang et al., 2020). Antibodies may be more effective when targeting EPS components that are more exposed or accessible on the biofilm surface. This approach can help overcome the penetration problem while still providing a degree of specificity (Jiang et al., 2020). The specification and size of an antibody can also affect its ability to penetrate biofilms. Smaller antibody fragments or engineered variants may have better permeability compared to full-size antibodies (Mazor et al., 2017). Antibody advancement against EPS components has promise for tackling certain biofilm infections and related antibiotic resistance, but their ability to penetrate and disperse biofilms adequately remains a challenge. Joint design strategy, carefully selected target and antibody form may help to solve these limitations, and improve its effectiveness in biofilm process.

#### 4.2.3 Inducing biofilm dispersion

Inducing biofilm dispersion is the process of breaking up or dispersing biofilms, which are organized populations of bacteria adhering to surfaces. It has been discovered to include EPS matrix degradation management, providing a research route for improving biofilm self-degradation.

The cyclic-di-GMP (c-di-GMP) is a secondary messenger nucleotide that plays a vital part in the biofilm lifecycle of both gram-positive and gram-negative bacteria, with higher c-di-GMP levels promoting biofilm formation and lower levels promoting biofilm disassembly (Jenal et al., 2017). Cyclic-di-GMP regulate the production of different enzymes, polysaccharides, and adhesion molecules involved with EPS (Peng et al., 2016), all of which are critical components that may be disturbed or inhibited to disrupt or inhibit EPS (Mann and Wozniak, 2012; Teschler et al., 2015). Thereby, inducing the decrease of c-di-GMP to inhibit the synthesis of EPS and induce biofilm dispersion is an important measure for the treatment of pathogen infection. *P. aeruginosa* has been extensively studied as a model bacterium for studying bacterial biofilms (K. Lee and Yoon, 2017). At the transcriptional level, Pel and Psl exopolysaccharides are favorably modulated by c-di-GMP (V. T. Lee et al., 2007). The transcriptional regulator FleQ acts as a receptor for c-di-GMP, and binding to c-di-GMP increases Pel and Psl transcription (Hickman and Harwood, 2008). FleQ forms a compound with the ATP-binding protein FleN in the absence of c-di-GMP, binding to the upper and lower sites of the Pel promoter. This combination promotes DNA bending, limiting transcription and biofilm development. In the presence of c-di-GMP, DNA remains unbent, triggering Pel transcription. Inhibiting c-di-GMP production enzymes or promoting c-di-GMP breakdown enzymes reduces c-di-GMP levels efficiently. Nitric oxide (NO) is a well-known approach for modifying c-di-GMP levels since it has been demonstrated to fine-tune c-di-GMP levels at low concentrations, promoting the dispersion of *P. aeruginosa* biofilms. This behavior has also been verified in numerous other bacterial species (Barraud et al., 2015). *Bacillus subtilis, S. aureus, S. pyogenes, Stench pseudomonas* also through a variety of mechanisms to reduce the c-di-GMP standards, and then destroy biofilms.

It is vital to realize that encouraging biofilm dispersion can have both beneficial and detrimental outcomes. While dispersion can aid in the management of biofilm-associated diseases and the prevention of new surface colonization, it can also result in the release of bacteria into the surrounding environment, potentially leading to systemic infection or inflammatory reactions (Rendueles and Ghigo, 2012). A greater amount of research is needed to investigate and refine approaches for inducing biofilm dispersion, taking into account the individual properties of the biofilm and the intended goal.

### 4.3 Enhancing biofilm control: physical disruption and combined approaches

**Figure 5:**
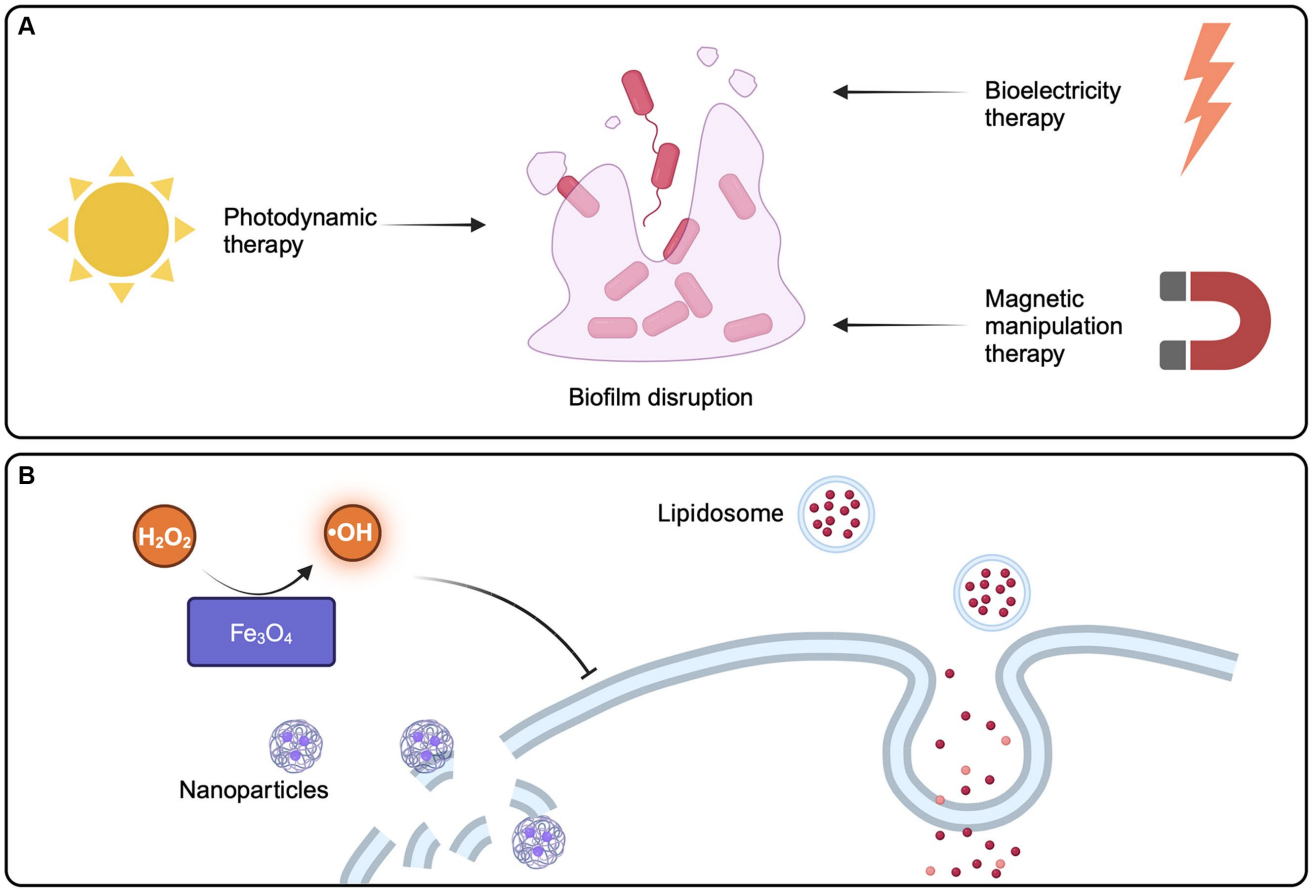
Strategies of physical methods and emerging technologies for disrupting pathogen biofilms. **(A)** Emerging technologies aimed at physically eliminating biofilms, such as photodynamic therapy, bioelectricity therapy, and magnetic manipulation therapy, have the potential to enhance and advance strategies for tackling biofilm-related issues. **(B)** Nanoparticle technology provides a new option to reduce the virulence of pathogenic bacteria biofilm. Created with BioRender.com.

Photodynamic therapy, bioelectricity therapy, and magnetic manipulation therapy all contribute to improved tactics for fighting intestinal EPS-based therapies (Figure 5).

In photodynamic therapy (PDT), non-toxic photosensitizers, such as tetrapyroroles, are irradiated with a light source of specific wavelength to produce reactive oxygen species (ROS), which can have lethal effects on microorganisms by destroying biofilms. Photosensitizers preferentially bind and accumulate in microbial cells without causing any damage to the host tissue. This approach is considered safe because it is nontoxic and minimally invasive, making it a reliable, realistic, and promising therapeutic strategy for reducing microbial burden and biofilm formation in chronic infections (Warrier et al., 2021).

Bioelectricity therapy, had a lot of research in the past 20 years, can now in the lab for *E. coli* in biological membrane damage, and fully support theory is used to eliminate the biofilm in the body (Kim et al., 2015). In addition, called Magnetic Manipulation Systems have been used in the treatment of *helicobacter pylori* biofilms (Yang et al., 2020). After exposed to the magnetic field in the body, can quickly penetrate biofilms, and quickly continuously release of antibiotics, prolonged antibiotic retained in the body (Yang et al., 2020).

These findings point out that physical removal approaches, such as photodynamic therapy, bioelectricity therapy, and magnetic manipulation therapy, may improve the curative effect of intestinal pathogenic bacteria biofilm resistance strategy. Considering the intricate process of biofilm formation and the complex interplay of microorganisms within their environments, a combined approach involving these approaches may be necessary to properly combat biofilm-mediated illnesses.

### 4.4 Application of novel technologies

Emerging technologies and bio/nanomaterials provide fresh options for minimising the deleterious effects of EPS-mediated biofilms on the host, particularly in the context of intestinal infections (Figure 5). Once in the pathogenic microbial environment, sustained-release organic medicines exhibit biocompatibility, penetrating biofilm interiors. Following medication delivery, EPS and internal microbial activity or metabolism are altered (Karygianni et al., 2020). In addition, liposomes, which are physiologically compatible vesicles composed of one or more phospholipid bilayers, are one of the most widely developed organic nanoparticles for drug delivery. They penetrate biofilms well and show potency against biofilms of a variety of bacteria (Rukavina and Vanić, 2016). Inorganic metal nanoparticles, such as silver, copper, iron oxide, and gold, can also be utilised as biofilm-targeting agents or nano-coatings (Koo et al., 2017), with which strengthen anti-biofilm efficiency while decreasing host toxicity, such as iron oxide (Fe_3_O_4_) exhibiting peroxidase-like activity, which catalyzes hydrogen peroxide (H_2_O_2_) to create hydroxyl radicals in a dose- and pH-dependent manner, allowing for the rapid elimination of internal biofilm infections (Gao et al., 2016). Gold NP can be used to treat *Vibrio cholerae*, providing a new method for the treatment of cholera (Kumar et al., 2023). What’s more, various polymeric nanoparticles have been developed to target and disrupt biofilms in the gastrointestinal tract. These nanoparticles can be designed to penetrate the EPS matrix and deliver antimicrobial agents directly to the bacterial cells within the biofilm (Thambirajoo et al., 2021). Nanoparticles can also be functional with specific molecules, in order to enhance its ability to target and destroy biofilms. For example, nanoparticles modified with enzymes that degrade EPS components can effectively weaken biofilm structures in the gut environment (Karnwal et al., 2023). Materials such as graphene oxide and carbon nanotubes have also shown potential in combating biofilms. These nanomaterials can interact with the EPS matrix and bacterial cells, potentially disrupting biofilm formation in the intestines (Kumar et al., 2023).

When targeting intestinal biofilms, it’s crucial to consider the specific challenges of the gastrointestinal environment, such as pH variations, enzymatic activity, and the presence of the gut microbiome. Nanobiomaterials designed for this purpose should be able to withstand these conditions and selectively target pathogenic biofilms without disrupting the beneficial gut microbiota. The use of nanobiomaterials to disrupt EPS-mediated biofilms in the intestines offers several advantages, including improved penetration of the biofilm matrix, targeted delivery of antimicrobial agents, and potential reduction in the development of antibiotic resistance. However, further research is needed to optimize these approaches and ensure their safety and efficacy in clinical applications.

## 5 Limitation of biofilm EPS in gut

Despite the fact that EPS in biofilms performs crucial part in numerous aspects of biofilm formation and function in the gut, the limitations of EPS in gut biofilms must not be overlooked. EPS can mediate both bacterial cohesion as well as biofilm adherence to surfaces (Muhammad et al., 2020).The interactions enabled by EPS, on the other hand, maybe particular to certain bacteria or situations and may not apply consistently to all gut biofilms. Bacterial adhesion to gastrointestinal tissues can be increased when EPS generation is inhibited (Dertli et al., 2015). While this may appear to be beneficial, it can also render germs less resistant. EPS can function as a barrier, preventing antimicrobial agents from diffusing and thereby limiting penetration into the biofilm’s core layers This presents challenges in effectively treating biofilm-associated gut infection (Karygianni et al., 2020). More research is needed to completely understand the complexities and dynamics of EPS in gut biofilms, as well as its implications for gut health.

## 6 Conclusion and prospect

EPS play a dual role in the gut microbiome: on one hand, EPS contribute to beneficial effects such as enhancing intestinal barrier function, supporting probiotic bacteria, and modulating immune responses. On the other hand, EPS produced by pathogenic bacteria can lead to detrimental outcomes. They facilitate the formation of robust biofilms, which are challenging to eradicate and can increase bacterial resistance to antibiotics. Excessive EPS production can protect pathogens from host immune responses and lead to chronic infections. Therefore, it is crucial to regulate the production and activity of EPS components in order to modify intestinal pathogenicity. To treat EPS from intestinal pathogen biofilms, several therapeutic strategies have been put forth. While targeting EPS in pathogenic biofilms holds promise for mitigating infections, it is essential to balance these interventions with the preservation of beneficial microbial functions. Therapeutic strategies must be tailored to the specific contexts of both harmful and beneficial EPS roles. Thus, more in-depth exploration of how the gut microbiome responds to these approaches is needed. In addition, the safety of these approaches is critical and must be thoroughly considered.

## Author contributions

FG: Conceptualization, Formal analysis, Writing – original draft. SX: Software, Validation, Writing – original draft. XL: Formal analysis, Visualization, Writing – original draft. CH: Formal analysis, Software, Writing – original draft. XY: Conceptualization, Visualization, Writing – original draft. LP: Formal analysis, Visualization, Writing – original draft. SZ: Resources, Software, Writing – original draft. HG: Supervision, Validation, Writing – review & editing. JX: Funding acquisition, Project administration, Supervision, Writing – review & editing.

## Funding

The author(s) declare that financial support was received for the research, authorship, and/or publication of this article. This work was funded by the National Natural Science Foundation of China Grant (No. 82174056 JX). The funder played no role in the review design, data collection, analysis and interpretation of data, as well as the writing of this manuscript.

## Conflict of interest

The authors declare that the research was conducted in the absence of any commercial or financial relationships that could be construed as a potential conflict of interest.
